# Supplementary material for: Integrated approach of brachial-ankle pulse wave velocity and cardiovascular risk scores for predicting the risk of cardiovascular events
Source: PLoS One. 2022 Apr 27;17(4):e0267614. doi: 10.1371/journal.pone.0267614 (PMC9045657; doi:10.1371/journal.pone.0267614)

PLOS ONE

Integrated Approach of Brachial-Ankle Pulse Wave Velocity and Cardiovascular Risk Scores for Predicting the Risk of Cardiovascular Events

--Manuscript Draft--

**Manuscript Number:**	PONE-D-22-03869
**Article Type:**	Research Article
**Full Title:**	Integrated Approach of Brachial-Ankle Pulse Wave Velocity and Cardiovascular Risk Scores for Predicting the Risk of Cardiovascular Events
**Short Title:**	baPWV and risk scores for cardiovascular events
**Corresponding Author:**	Hack-Lyoung Kim<br>SMG-SNU Boramae Medical Center<br>Seoul, Seoul KOREA, REPUBLIC OF
**Keywords:**	arterial stiffness; cardiovascular risk score; prognosis; pulse wave velocity.
**Abstract:**	Background<br>The 2013 American College of Cardiology (ACC)/American Heart Association (AHA) atherosclerotic cardiovascular disease (ASCVD) risk score may be insufficient for accurate prediction in Asians. This study was performed to investigate whether brachial-ankle pulse wave velocity (baPWV) has additional prognostic value to the risk score estimated by the ACC/AHA pooled cohort equations (PCEs).<br>Methods It needs correction<br>A total of 6,359 patients (3,534 men and 2,825 women) aged 40-9 years without documented cardiovascular disease who underwent baPWV measurement were retrospectively analyzed. Cardiovascular risk scores were calculated using the 2013 ACC/AHA PCEs. Cardiovascular events, including cardiac death, non-fatal myocardial infarction, coronary revascularization and ischemic stroke, were assessed.<br>Results<br>During a median follow-up period of 4.0 years (interquartile range 1.7-6.1 years), cardiovascular events occurred in 129 patients (2.0%). The receiver operating characteristic curve analysis showed that baPWV was stronger in the detection of cardiovascular events than the 2013 ACC/AHA risk score (area under the curve: 0.70 versus 0.62, *p* <0.001). In the multivariable Cox regression analysis, both baPWV and 2013 ACC/AHA risk score were independently associated with the occurrence of clinical events (*p* <0.001 for each). The baPWV had incremental prognostic value to the 2013 ACC/AHA risk score in predicting clinical events (global chi-square from 21.23 to 49.51, *p* <0.001).<br>Conclusion<br>The baPWV appears to be a strong predictor of the risk of cardiovascular events in Koreans. Measuring baPWV in addition to the 2013 ACC/AHA risk score helps identify individuals aged 40-79 years without previous cardiovascular diseases.
**Order of Authors:**	Hyue Mee Kim
	Tae-Min Rhee
	Hack-Lyoung Kim
**Additional Information:**	
**Question**	**Response**
**Financial Disclosure**<br><br>Enter a financial disclosure statement that describes the sources of funding for the work included in this submission. Review the submission guidelines for detailed requirements. View published research articles from *PLOS ONE* for specific	The authors received no specific funding for this work.

Powered by Editorial Manager® and ProduXion Manager® from Aries Systems Corporation

examples.<br><br>This statement is required for submission and **will appear in the published article** if the submission is accepted. Please make sure it is accurate.<br><br>**Unfunded studies**<br>Enter: *The author(s) received no specific funding for this work.*<br><br>**Funded studies**<br>Enter a statement with the following details:<br>• Initials of the authors who received each award<br>• Grant numbers awarded to each author<br>• The full name of each funder<br>• URL of each funder website<br>• Did the sponsors or funders play any role in the study design, data collection and analysis, decision to publish, or preparation of the manuscript?<br>• **NO** - Include this sentence at the end of your statement: *The funders had no role in study design, data collection and analysis, decision to publish, or preparation of the manuscript.*<br>• **YES** - Specify the role(s) played.<br><br>* typeset	
**Competing Interests**<br><br>Use the instructions below to enter a competing interest statement for this submission. On behalf of all authors, disclose any competing interests that could be perceived to bias this work—acknowledging all financial support and any other relevant financial or non-financial competing interests.<br><br>This statement is required for submission and **will appear in the published article** if the submission is accepted. Please make sure it is accurate and that any funding sources listed in your Funding Information later in the submission form are also declared in your Financial Disclosure statement.	The authors have declared that no competing interests exist.

View published research articles from *PLOS ONE* for specific examples.<br><br>**NO authors have competing interests**<br><br>Enter: *The authors have declared that no competing interests exist.*<br><br>**Authors with competing interests**<br><br>Enter competing interest details beginning with this statement:<br><br>*I have read the journal's policy and the authors of this manuscript have the following competing interests: [insert competing interests here]*<br><br>* typeset	
**Ethics Statement**<br><br>Enter an ethics statement for this submission. This statement is required if the study involved:<br><br>• Human participants<br>• Human specimens or tissue<br>• Vertebrate animals or cephalopods<br>• Vertebrate embryos or tissues<br>• Field research<br><br>Write "N/A" if the submission does not require an ethics statement.<br><br>General guidance is provided below. Consult the submission guidelines for detailed instructions. **Make sure that all information entered here is included in the Methods section of the manuscript.**	The study was carried out according to the principles of the Declaration of Helsinki and approved by the Clinical Research Institute (IRB) of Seoul National University Boramae Medical Center (Seoul, South Korea) (IRB No.10-2020-34). Informed consent was waived by the IRB due to its retrospective study design and routine nature of information collected.

Powered by Editorial Manager® and ProduXion Manager® from Aries Systems Corporation

**Format for specific study types**<br><br>**Human Subject Research (involving human participants and/or tissue)**<br>• Give the name of the institutional review board or ethics committee that approved the study<br>• Include the approval number and/or a statement indicating approval of this research<br>• Indicate the form of consent obtained (written/oral) or the reason that consent was not obtained (e.g. the data were analyzed anonymously)<br><br>**Animal Research (involving vertebrate animals, embryos or tissues)**<br>• Provide the name of the Institutional Animal Care and Use Committee (IACUC) or other relevant ethics board that reviewed the study protocol, and indicate whether they approved this research or granted a formal waiver of ethical approval<br>• Include an approval number if one was obtained<br>• If the study involved *non-human primates*, add *additional details* about animal welfare and steps taken to ameliorate suffering<br>• If anesthesia, euthanasia, or any kind of animal sacrifice is part of the study, include briefly which substances and/or methods were applied<br><br>**Field Research**<br><br>Include the following details if this study involves the collection of plant, animal, or other materials from a natural setting:<br>• Field permit number<br>• Name of the institution or relevant body that granted permission	
**Data Availability**<br><br>Authors are required to make all data underlying the findings described fully available, without restriction, and from the time of publication. PLOS allows rare exceptions to address legal and ethical concerns. See the PLOS Data Policy and FAQ for detailed information.	Yes - all data are fully available without restriction

Powered by Editorial Manager® and ProduXion Manager® from Aries Systems Corporation

A Data Availability Statement describing where the data can be found is required at submission. Your answers to this question constitute the Data Availability Statement and **will be published in the article**, if accepted.<br><br>**Important:** Stating 'data available on request from the author' is not sufficient. If your data are only available upon request, select 'No' for the first question and explain your exceptional situation in the text box.<br><br>Do the authors confirm that all data underlying the findings described in their manuscript are fully available without restriction?	
**Describe where the data may be found in full sentences. If you are copying our sample text, replace any instances of XXX with the appropriate details.**<br><br>• If the data are **held or will be held in a public repository**, include URLs, accession numbers or DOIs. If this information will only be available after acceptance, indicate this by ticking the box below. For example: *All XXX files are available from the XXX database (accession number(s) XXX, XXX.*).<br>• If the data are all contained **within the manuscript and/or Supporting Information files**, enter the following: *All relevant data are within the manuscript and its Supporting Information files.*<br>• If neither of these applies but you are able to provide **details of access elsewhere**, with or without limitations, please do so. For example:<br><br>*Data cannot be shared publicly because of [XXX]. Data are available from the XXX Institutional Data Access / Ethics Committee (contact via XXX) for researchers who meet the criteria for access to confidential data.*<br><br>*The data underlying the results presented in the study are available from (include the name of the third party*	All relevant data are within the manuscript

and contact information or URL).<br>• This text is appropriate if the data are owned by a third party and authors do not have permission to share the data.<br><br>* typeset	
Additional data availability information:	

Powered by Editorial Manager® and ProduXion Manager® from Aries Systems Corporation

Manuscript
Click here to access/download;Manuscript;Plos one _clear file.docx

Integrated Approach of Brachial-Ankle Pulse Wave Velocity and Cardiovascular Risk Scores for Predicting the Risk of Cardiovascular Events

Short title: baPWV and risk scores for cardiovascular events

Hyue Mee Kim,[1] Tae-Min Rhee,[2] Hack-Lyoung Kim[2]

[1]Division of Cardiology, Department of Internal Medicine, Chung-Ang University Hospital, Chung-Ang University College of Medicine, Seoul, South Korea

[2]Division of Cardiology, Department of Internal Medicine, Boramae Medical Center, Seoul National University College of Medicine, Seoul, South Korea

Corresponding author:

Hack-Lyoung Kim, MD, PhD,

Division of Cardiology, Department of Internal Medicine, Boramae Medical Center, Seoul National University College of Medicine, Seoul, South Korea

E-mail: khl2876@gmail.com

Abstract

Background

The 2013 American College of Cardiology (ACC)/American Heart Association (AHA) atherosclerotic cardiovascular disease (ASCVD) risk score may be insufficient for accurate prediction in Asians. This study was performed to investigate whether brachial-ankle pulse wave velocity (baPWV) has additional prognostic value to the risk score estimated by the ACC/AHA pooled cohort equations (PCEs).

Methods

A total of 6,359 patients (3,534 men and 2,825 women) aged 40-9 years without documented cardiovascular disease who underwent baPWV measurement were retrospectively analyzed. Cardiovascular risk scores were calculated using the 2013 ACC/AHA PCEs. Cardiovascular events, including cardiac death, non-fatal myocardial infarction, coronary revascularization and ischemic stroke, were assessed.

Results

During a median follow-up period of 4.0 years (interquartile range 1.7-6.1 years), cardiovascular events occurred in 129 patients (2.0%). The receiver operating characteristic curve analysis showed that baPWV was stronger in the detection of cardiovascular events than the 2013 ACC/AHA risk score (area under the curve: 0.70 versus 0.62, *p*<0.001). In the multivariable Cox regression analysis, both baPWV and 2013 ACC/AHA risk score were independently associated with the occurrence of clinical events (*p* <0.001 for each). The baPWV had incremental prognostic value to the 2013 ACC/AHA risk score in predicting clinical events (global chi-square from 21.23 to 49.51, *p*<0.001).

Conclusion

Line 4 needs more clarity. Predicting " MACE"

Line 9 needs correction - "40-79 years "

The baPWV appears to be a strong predictor of the risk of cardiovascular events in Koreans. Measuring baPWV in addition to the 2013 ACC/AHA risk score helps identify individuals aged 40-79 years without previous cardiovascular diseases.

Line 25 needs more information. " Helps identify individuals at risk for MACE"

Keywords: arterial stiffness; cardiovascular risk score; prognosis; pulse wave velocity.

Introduction

Atherosclerotic cardiovascular disease (ASCVD), including coronary heart disease and ischemic stroke, constitutes a major public health problem, showing high prevalence and mortality worldwide.[1] Controlling modifiable risk factors, such as hypertension, diabetes mellitus, dyslipidemia, smoking, physical inactivity and obesity, can effectively prevent the development of ASCVD.[2, 3] Additionally, it has been suggested that the benefits of risk management are great in individuals at high risk.[4] Therefore, early screening of high-risk patients and customized management are advantageous in terms of improving prognosis.

To predict ASCVD risk, many investigators have developed several risk-predicting algorithms.[5-7] In 2013, the American Heart Association (AHA) and the American College of Cardiology (ACC) announced a new ASCVD risk score calculated from age, sex, race, cholesterol levels, blood pressure, diabetes, smoking status and medication, to guide ASCVD risk-reducing treatments.[4] However, the applicability of this risk score to other races has been questioned, because it has been found to overestimate or underestimate the risk in several populations including East Asians.[8-11]

Arterial stiffness, another important risk-predicting marker that reflects structural and functional changes in the arterial wall, is associated with the development of cardiovascular events independent of traditional risk factors.[12-15] Among various measures of arterial stiffness, brachial-ankle pulse wave velocity (baPWV) has recently been widely used due to its non-invasiveness and simplicity.[16] Moreover, clinical usefulness of baPWV has been validated in many studies.[17-21]

Previously, our study group reported significant correlations between baPWV and several cardiovascular risk scores including 2013 ACC/AHA ASCVD risk score.[22] The aim of this

study is to compare prognostic value between baPWV and 2013 ACC/AHA ASCVD risk score, and to determine whether baPWV has incremental value to 2013 ACC/AHA ASCVD risk score in predicting future cardiovascular events.

Methods

Study population

We retrospectively identified consecutive individuals who visited cardiovascular center of a general hospital in a big city (Seoul, South Korea) and underwent baPWV measurement as part of the cardiovascular examination between January 2010 and June 2018. A total of 11,767 individuals who were screened for the study. Among the individuals, we excluded the following patients: (1) those with a history of previous ASCVD, (2) those aged <40 years or >79 years, (3) those with missing information for calculating cardiovascular risk, and (4) those with a low ankle-brachial index (ABI <0.9) score. A total of 6,359 individuals were included in the final analysis. The study was carried out according to the principles of the Declaration of Helsinki and approved by the Clinical Research Institute (IRB) of Seoul National University Boramae Medical Center (Seoul, South Korea) (IRB No.10-2020-34). Informed consent was waived by the IRB due to its retrospective study design and routine nature of information collected.

Clinical data collection

Body mass index (BMI) was calculated as weight (kg) divided by the square of the height (m^2). BMI >25 kg/m^2 was defined as obesity.[23] Systolic and diastolic blood pressures (SBP and DBP) were measured using an oscillometric device by a trained nurse. Hypertension was

defined based on previous diagnosis, current use of anti-hypertensive medications or SBP/DBP ≥ 140/90 mmHg. Diabetes mellitus was defined based on previous diagnosis, current use of anti-diabetic medications or fasting glucose ≥ 126 mg/dL. Dyslipidemia was defined based on previous diagnosis or low-density lipoprotein (LDL) cholesterol level ≥ 160 mg/dL.[24] Individuals were classified as smokers if they had smoked regularly during the previous 12 months. After overnight fasting, venous blood sample was obtained and blood levels of the following parameters were analyzed: total cholesterol, LDL cholesterol, high-density lipoprotein (HDL) cholesterol, triglyceride, C-reactive protein, hemoglobin, glycated hemoglobin and creatinine. Estimated glomerular filtration rate (GFR) was obtained by the Modification of Diet in Renal Disease (MDRD) equation. Concomitant cardiovascular medications were also assessed, which included antiplatelets, calcium channel blockers, renin-angiotensin system blockers, beta-blockers, and statin.

baPWV measurement

We measured baPWV noninvasively using validated protocols that have previously been published.[18, 20] Briefly, caffeine ingestion and cigarette smoking were not allowed before the measurement; however, regular medication was continued. After the patient was placed in the supine position for 5 or more minutes, blood pressure and baPWV were automatically generated using a noninvasive automated waveform analyzer (VP-1000; Colin Co., Ltd., Komaki, Japan). Then, baPWV was measured in upper and lower extremities with a plethysmographic sensor, which simultaneously recorded blood pressure, electrocardiogram, and heart sounds. The baPWV was calculated by dividing the distance by the transit time, and the distance between measurement points was estimated by individual height. The transit

How many hours prior?

time was calculated from the starting point of the brachial pulse wave to the start of the ankle pulse wave. The mean values of right-sided and left-sided baPWV measurements were used for the analysis. All measurements were taken by an experienced operator, and the intra-observer coefficient of variation was approximately 5.1% in our laboratory.[20]

Calculation of the ACC/AHA risk scores

We calculated the 10-year ASCVD risk using the 2013 ACC/AHA pooled cohort equations (PCEs).[4] We used sex-specific equations for non-Hispanic whites because there was no specific equation for Koreans. Risk scores were calculated using the following parameters: age, sex, hypertension, diabetes mellitus, smoking, total and HDL cholesterol and blood pressure. Cardiovascular risks of study subjects were classified according to the ACC/AHA risk scores: low risk, <5%; borderline risk, 5%-7.4%; intermediate risk, 7.5%-19.9%; or high risk, ≥20%.[25]

Assessment of cardiovascular events

Data on clinical events were obtained from hospital records described by physicians, telephone contracts or national death data. The primary endpoint of this study was a composite of the occurrence of cardiac death, non-fatal myocardial infarction, coronary revascularization and ischemic stroke. Cardiac death was defined as one after acute coronary syndrome, pump failure, stroke or sudden death. Non-fatal myocardial infarction was defined based on electrocardiographic findings, elevated cardiac enzyme levels and coronary angiography results. Coronary revascularization included percutaneous coronary intervention and/or coronary bypass surgery. Ischemic stroke was diagnosed by typical neurological signs

Consider replacing pump failure with HFrEF and sudden death with sudden cardiac death

and symptoms that were assessed by neurologists and noninvasive brain imaging findings, such as brain computed tomography and/or magnetic resonance imaging.

Statistical analysis

Continuous variables are expressed as means ± standard deviations, and categorical variables are expressed as numbers and percentages. Differences between continuous variables were compared using Student's *t* test for independent variables, and differences between categorical variables were compared using Pearson's chi-square test. The cutoff value of baPWV to predict cardiovascular events was set according to the receiver operating characteristic (ROC) curve analysis with the highest sum of sensitivity and specificity. ROC curves were generated to compare the predictive value of baPWV and the ACC/AHA risk score. Comparisons of the areas under the curve (AUCs) were conducted using Delong's method.[26] Event rates were estimated using event counts and exposure over time. Cox proportional hazard regression analyses were performed to evaluate the predictive values of baPWV and ACC/AHA risk score. The following clinical covariates were considered potential confounders and included as confounding variables in the multivariable analysis. The hazard ratios (HRs) and 95% confidence intervals (CIs) were calculated. Event-free survival analyses were conducted using the Kaplan–Meier method with the log-rank test and Cox proportional hazard model. All statistical analyses were performed using SPSS (version 22.0; SPSS Inc., Chicago, IL, USA), and statistical significance was set at $p<0.05$.

Results

Baseline characteristics

The baseline characteristics of the study population are summarized in **Table 1**. The mean age of the study population was 59.9±8.8 years, and 55.6% of the patients were male. The prevalence rates of hypertension, diabetes mellitus, dyslipidemia and current smoking among the patients were 43.4%, 22.7%, 48.6%, and 13.8%, respectively. Approximately half of the patients (47.6%) had obesity. Major laboratory examination findings, including cholesterol, hemoglobin, and C-reactive protein levels and the GFR, were within the normal range. A total of 28.4% of the individuals took a renin-angiotensin system blocker, and 18.6% were prescribed beta-blockers. The proportion of patients taking antiplatelet drugs, calcium channel blockers and statins were 21.9%, 22.3% and 44.5%, respectively.

Comparison between patients with and without clinical events

During a median follow-up period of 4.0 years (interquartile range 1.7-6.1 years), 129 cardiovascular events, including cardiovascular death (n=10), non-fatal myocardial infarction (n = 2), coronary revascularization (n = 69) and ischemic stroke (n = 48), occurred. Comparisons of baseline characteristics between individuals with and without events are shown in **Table 1**. Patients with cardiovascular events were older and had more cardiovascular risk factors, including hypertension, diabetes mellitus, dyslipidemia and current smoking, compared to those without. Total cholesterol and LDL cholesterol levels were lower in patients with events than those without, and the other laboratory findings, including HDL cholesterol, triglyceride, and HbA1c levels and the GFR, were not significantly different between the 2 groups. The proportion of patients taking antiplatelet agents, beta-blockers and statins were higher in patients with events than in those without.

Table 1. Baseline characteristics of study population

Characteristic	Total (n=6359)	Subjects with events (n=129)	Subjects without events (n=6230)	*p* value
Age, years	59.9±8.8	62.2±7.9	59.9±8.8	0.003
Male, sex	3534 (55.6)	78 (60.5)	3456 (55.5)	0.259
Body mass index, kg/m^2	25.0±3.3	25.0±3.0	25.0±3.3	0.815
Systolic blood pressure, mmHg	129.9±17.7	130.9±19.6	130.0±17.7	0.534
Diastolic blood pressure, mmHg	78.3±11.0	76.8±10.8	78.4±11.1	0.124
Heart rate, per min	69.5±11.8	68.5±11.7	69.5±11.8	0.400
Risk factors				
Hypertension	2758 (43.4)	73 (56.6)	2685 (43.1)	0.002
Diabetes mellitus	1444 (22.7)	49 (38.0)	1395 (22.4)	<0.001
Dyslipidemia	3088 (48.6)	90 (69.8)	2998 (48.1)	<0.001
Smoking	878 (13.8)	34 (26.4)	844 (13.5)	<0.001
Obesity (body mass index ≥25kg/m^2)	3025 (47.6)	58 (45.0)	2967 (47.6)	0.548
Laboratory findings				
Total cholesterol, mg/dL	164.2±37.5	152.5±38.6	164.4±37.4	<0.001
Low-density lipoprotein cholesterol, mg/dL	94.1±33.5	83.8±36.0	94.3±33.4	<0.001
High-density lipoprotein cholesterol, mg/dL	49.9±12.8	47.5±14.6	50.0±12.8	0.032
Triglyceride, mg/dL	129.2±79.5	126.2±73.7	129.2±79.7	0.657
C-reactive protein, mg/dL	0.7±2.9	1.8±5.0	0.7±2.8	<0.001
Hemoglobin, g/dL	13.8±1.7	13.3±1.8	13.8±1.7	0.005
Glycated hemoglobin, %	6.4±1.1	6.6±1.2	6.4±1.1	0.053
Glomerular filtration rate, mL/min/1.73m^2	87.8±23.7	84.6±27.8	87.9±23.6	0.127
Concomitant medications				
Antiplatelet	1394 (21.9)	48 (37.2)	1346 (21.6)	<0.001
Calcium channel blocker	1417 (22.3)	31 (24.0)	1386 (22.2)	0.630
RAS blocker	1804 (28.4)	46 (35.7)	1758 (28.2)	0.064
Beta-blocker	1184 (18.6)	43 (33.3)	1141 (17.9)	<0.001
Statin	2831 (44.5)	78 (60.5)	2753 (44.2)	<0.001

Numbers are expressed as mean±SD or n (%). RAS: renin-angiotensin system

Comparison of the predictive value between the ACC/AHA risk score and baPWV

The predictive value for cardiovascular events was compared between ACC/AHA risk score and baPWV using ROC curve analyses (**Fig 1**). The AUC of the ACC/AHA risk score and baPWV for predicting cardiovascular events were 0.62 (95% CI, 0.61-0.64, p <0.001) and 0.70 (95%, CI 0.69-0.71, p <0.001), respectively. Pairwise comparisons of the ROC curve showed that baPWV was more powerful in predicting cardiovascular events than the ACC/AHA risk score (p <0.001) (**Table 2**). In ROC curve analysis, the cutoff value of baPWV (=1556 cm/s) for the best prediction of cardiovascular events was obtained. In Kaplan-Meier survival analysis, event-free survival rates were significantly different according to the cutoff values of baPWV and ACC/AHA risk scores (log-rank p <0.001 for each) **(Fig 2).** In multivariable Cox regression analysis, patients with an intermediate ACC/AHA ASCVD risk (HR, 2.09; 95% CI, 1.32-3.30; p =0.002) and a high ACC/AHA ASCVD risk (HR, 3.02; 95% CI, 1.82-5.00; p <0.001) had higher cardiovascular event rates compared to those with low ACC/AHA ASCVD risk. A higher baPWV (≥1,556 cm/s) was also significantly associated with an increased risk of cardiovascular events in the same multivariable analysis (HR, 3.75; 95% CI, 2.34-6.00; p <0.001) (**Table 3**).

Fig 1. **The receiver operating characteristic analysis showing the prognostic value of ACC/AHA ASCVD risk score and baPWV**

ACC/AHA ASCVD, American College of Cardiology/American Heart Association atherosclerotic cardiovascular disease; baPWV, brachial-ankle pulse wave velocity.

Fig 2. Event-free survival rates according to ACC/AHA ASCVD risk score (A) and baPWV (B)

ACC/AHA ASCVD, American College of Cardiology/American Heart Association atherosclerotic cardiovascular disease; baPWV, brachial-ankle pulse wave velocity.

Additional prognostic value of baPWV to the ACC/AHA risk score

A higher baPWV (≥1,556 cm/s) provided incremental prognostic value to the ACC/AHA risk score in the prediction of ASCVD (global chi-square score, from 21.23 to 49.51, p <0.001) (**Fig 3**).

Fig 3. The additive prognostic value of baPWV

ACC/AHA, American College of Cardiology/American Heart Association; baPWV, brachial-ankle pulse wave velocity.

Table 2. Receiver operating characteristic curve analyses

**Parameter**	**AUC**	**95% CI**	***p* value**	**Comparison with DeLong**
2013 ACC/AHA ASCVD risk score	0.62	0.61-0.63	<0.001	Ref.
baPWV	0.70	0.68-0.71	<0.001	0.001

2013 ACC/AHA ASVD risk score: 2013 American College of Cardiology/American Heart Association atherosclerotic cardiovascular disease risk score, baPWV: brachial-ankle pulse wave velocity, AUC: area under the curve

Table 3. Prognostic values of 2013ACC/AHA risk score and baPWV in predicting cardiovascular events

**Risk**	**HR**	**95% CI**	***p* value**
2013 ACC/AHA ASCVD low risk (<5%)	1		
2013 ACC/AHA ASCVD Borderline risk (5-7.4%)	1.52	0.81-2.84	0.190
2013 ACC/AHA ASCVD Intermediate risk (7.5-19.99%)	2.09	1.32-3.30	0.002
2013 ACC/AHA ASCVD High risk (≥20%)	3.02	1.82-5.00	<0.001
baPWV≥1,556 cm/s (unadjusted)	3.58	2.41-5.31	<0.001
baPWV ≥1,556 cm/s (adjusted)*	3.75	2.34-6.00	<0.001

2013 ACC/AHA ASVD risk score: 2013 American College of Cardiology/American Heart Association atherosclerotic cardiovascular disease risk score, baPWV: brachial-ankle pulse wave velocity, HR: hazard ratio, CI: confidence interval

*Covariates were controlled as potential confounders: Age, Sex, BMI, HR, DM, HTN, Dyslipidemia, Smoking, Beta blockers, RAS blockers, Statin

Discussion

Our study demonstrated that both baPWV and the 2013 ACC/AHA risk score were independently associated with the development of future cardiovascular events in Korean subjects aged 40-79 years without prior history of documented cardiovascular disease. The baPWV showed a stronger association with the risk of cardiovascular events than the ACC/AHA risk score. In addition, high baPWV provided incremental prognostic value over the ACC/AHA risk score, regarding the prediction of cardiovascular events. To the best of our knowledge, this is the first study to show the prognostic value of baPWV compared to the ACC/AHA risk score. Furthermore, our findings suggest for the first time that an integrated approach of baPWV and ACC/AHA risk score could improve risk assessment.

The 2013 ACC/AHA PCEs were derived using data from 5 large epidemiological cohort studies conducted in the US (Atherosclerosis Risk in Communities, Cardiovascular Health Study, Coronary Artery Risk Development in Young Adults and the Framingham and Framingham Offspring studies); however, the data for the follow-up of Hispanic and Asian-American cohorts were limited.[4] Many countries in Asia and Europe have performed external validation of the risk score system, and they found overestimated or underestimated cardiovascular risk by the PCEs in each country.[8-11] Although several countries have developed their own risk prediction model for ASCVD,[27, 28] they are under-utilized, and the 2013 ACC/AHA PCEs is still widely used due to its accessibility. In this background, further testing to complement the ACC/AHA PCEs for appropriate risk prediction in these countries would be needed. Given our findings, we believe that non-invasive and easy to measure baPWV will be a suitable test for this purpose.

Arterial stiffness is the result of the degenerative process affected by aging and arteriosclerosis.[29, 30] Measuring arterial stiffness is clinically important because it provides prognostic implications for cardiovascular diseases.[12, 31] PWV is the most widely used measure of arterial stiffness.[16, 32] As PWV is a measure of the time it takes the pulse to travel between the 2 arterial points, it is a direct measure of arterial stiffness. PWV is proportional to the speed of pulse wave through the arterial walls: when the blood vessels are young and elastic, the speed of the pulse wave traveling along the arterial wall is low; otherwise, in stiffened artery, the PWV is high.[22] Although carotid-femoral pulse wave velocity is considered a gold standard non-invasive measure of arteria stiffness and has abundant clinical data.[16, 32] it has several shortcomings such as technical difficulty and inconvenience to the patients during the exam.[16] baPWV, a more recently developed method, is easier and simpler to measure, and is widely used in Asians.[16] The usefulness of baPWV has also been validated in many clinical studies[17-20] and meta-analyses.[33, 34] Measuring baPWV is recommended by the guidelines for the management of hypertension in Japan.[35] Considering its non-invasiveness and simplicity, baPWV may be a good risk stratification tool especially in mass screening.

Most newly eligible individuals who need risk stratification have no known cardiovascular diseases and are at low risk. Recently, achievement of various studies regarding ASCVD preventive effects of statins have broadened the spectrum of statins in primary prevention.[25] Therefore, it is clinically important to select individuals for whom the statins can be helpful by appropriating risk stratification considering the benefits and harm or side effects caused by long-term treatment of statins. In general, clinical risk assessment could improve when using 2 or more risk stratification tools rather than using 1 method. Previous studies showed that the addition of baPWV to clinical and laboratory

findings significantly increased the predictive power of future cardiovascular events.[13, 20, 36, 37] Additionally, we confirmed the additional predictive value of baPWV for ASCVD when it added to the ACC/AHA PCEs in this study. In line with our findings, coronary calcium scoring (CACS) have recently been recommended for helping guide decisions about preventive intervention in select individuals.[25] Given that CACS can reclassify estimated risk and modify treatment plan when it is added to risk scores, our study suggests that baPWV could also be used as an addictive tool to more meticulously stratify cardiovascular risk in primary prevention.

Study limitations

This study has several limitations. First, this was a single-center retrospective study; thus, there might be a selection bias. Secondly, as our data were from Koreans aged 40-79 years, generalizing the results in other races is challenging. Thirdly, we used the 2013 ACC/AHA PCEs for non-Hispanic whites; thus, there would be discrepancies in applying the algorithms to Koreans. Finally, baPWV is known to be affected by other cardiovascular risk factors. Although we excluded patients with a low ABI score and made corrections using the multivariable analysis, we could not completely rule out possible confounding factors.

Conclusions

The prognostic value of baPWV for future cardiovascular events in the Korean general population was superior to that of the 2013 ACC/AHA PCEs. Also, combined information on baPWV and the 2013 ACC/AHA PCEs showed additive predictive value. Our findings

It's a retrospective study. I do not recommend using " Superiority ".

suggest that an integrated approach of baPWV and the ACC/AHA risk scores could improve risk assessment and risk management.

Consider changing line 290.

References

1. Virani SS, Alonso A, Benjamin EJ, Bittencourt MS, Callaway CW, Carson AP, et al. Heart Disease and Stroke Statistics-2020 Update: A Report From the American Heart Association. Circulation. 2020;141(9):e139-e596. 2

2. Wong ND. Epidemiological studies of CHD and the evolution of preventive cardiology. Nature reviews Cardiology. 2014;11(5):276-89.

3. O'Donnell MJ, Chin SL, Rangarajan S, Xavier D, Liu L, Zhang H, et al. Global and regional effects of potentially modifiable risk factors associated with acute stroke in 32 countries (INTERSTROKE): a case-control study. Lancet (London, England). 2016;388(10046):761-75.

4. Goff DC, Jr., Lloyd-Jones DM, Bennett G, Coady S, D'Agostino RB, Gibbons R, et al. 2013 ACC/AHA guideline on the assessment of cardiovascular risk: a report of the American College of Cardiology/American Heart Association Task Force on Practice Guidelines. Circulation. 2014;129(25 Suppl 2):S49-73.

5. Wilson PW, D'Agostino RB, Levy D, Belanger AM, Silbershatz H, Kannel WB. Prediction of coronary heart disease using risk factor categories. Circulation. 1998;97(18):1837-47.

6. Third Report of the National Cholesterol Education Program (NCEP) Expert Panel on Detection, Evaluation, and Treatment of High Blood Cholesterol in Adults (Adult Treatment Panel III) final report. Circulation. 2002;106(25):3143-421.

7. D'Agostino RB, Sr., Vasan RS, Pencina MJ, Wolf PA, Cobain M, Massaro JM, et al. General cardiovascular risk profile for use in primary care: the Framingham Heart Study. Circulation. 2008;117(6):743-53.

8. DeFilippis AP, Young R, McEvoy JW, Michos ED, Sandfort V, Kronmal RA, et al. Risk score overestimation: the impact of individual cardiovascular risk factors and preventive therapies on the performance of the American Heart Association-American College of Cardiology-Atherosclerotic Cardiovascular Disease risk score in a modern multi-ethnic cohort. European heart journal. 2017;38(8):598-608.

9. Kavousi M, Leening MJ, Nanchen D, Greenland P, Graham IM, Steyerberg EW, et al. Comparison of application of the ACC/AHA guidelines, Adult Treatment Panel III guidelines, and European Society of Cardiology guidelines for cardiovascular disease prevention in a European cohort. Jama. 2014;311(14):1416-23.

10. Volgman AS, Palaniappan LS, Aggarwal NT, Gupta M, Khandelwal A, Krishnan AV, et al. Atherosclerotic Cardiovascular Disease in South Asians in the United States: Epidemiology, Risk Factors, and Treatments: A Scientific Statement From the American Heart Association. Circulation. 2018;138(1):e1-e34.

11. Hutchinson RN, Shin S. Systematic review of health disparities for cardiovascular diseases and associated factors among American Indian and Alaska Native populations. PloS one. 2014;9(1):e80973.

12. Mattace-Raso FU, van der Cammen TJ, Hofman A, van Popele NM, Bos ML, Schalekamp MA, et al. Arterial stiffness and risk of coronary heart disease and stroke: the Rotterdam Study. Circulation. 2006;113(5):657-63.

13. Mitchell GF, Hwang SJ, Vasan RS, Larson MG, Pencina MJ, Hamburg NM, et al. Arterial stiffness and cardiovascular events: the Framingham Heart Study. Circulation. 2010;121(4):505-11.

14. Stehouwer CD, Henry RM, Ferreira I. Arterial stiffness in diabetes and the metabolic syndrome: a pathway to cardiovascular disease. Diabetologia. 2008;51(4):527-39.

15. Guerin AP, Blacher J, Pannier B, Marchais SJ, Safar ME, London GM. Impact of aortic stiffness attenuation on survival of patients in end-stage renal failure. Circulation. 2001;103(7):987-92.

16. Kim HL, Kim SH. Pulse Wave Velocity in Atherosclerosis. Frontiers in cardiovascular medicine. 2019;6:41. Epub 2019/04/27.

17. Yamashina A, Tomiyama H, Takeda K, Tsuda H, Arai T, Hirose K, et al. Validity, reproducibility, and clinical significance of noninvasive brachial-ankle pulse wave velocity measurement. Hypertension research : official journal of the Japanese Society of Hypertension. 2002;25(3):359-64.

18. Kim HL, Im MS, Seo JB, Chung WY, Kim SH, Kim MA, et al. The association between arterial stiffness and left ventricular filling pressure in an apparently healthy Korean population. Cardiovascular ultrasound. 2013;11(1):2.

19. Kim HL, Lee JM, Seo JB, Chung WY, Kim SH, Zo JH, et al. The effects of metabolic syndrome and its components on arterial stiffness in relation to gender. Journal of cardiology. 2015;65(3):243-9.

20. Lee HS, Kim HL, Kim H, Hwang D, Choi HM, Oh SW, et al. Incremental Prognostic Value of Brachial-Ankle Pulse Wave Velocity to Single-Photon Emission Computed Tomography in Patients with Suspected Coronary Artery Disease. Journal of atherosclerosis and thrombosis. 2015;22(10):1040-50.

21. Sheng CS, Li Y, Li LH, Huang QF, Zeng WF, Kang YY, et al. Brachial-ankle pulse wave velocity as a predictor of mortality in elderly Chinese. Hypertension (Dallas, Tex : 1979). 2014;64(5):1124-30.

22. Lee CC, Tsai MC, Liu SC, Pan CF. Relationships between chronic comorbidities and the atherosclerosis indicators ankle-brachial index and brachial-ankle pulse wave velocity in

patients with type 2 diabetes mellitus. Journal of investigative medicine : the official publication of the American Federation for Clinical Research. 2018;66(6):966-72.

23. Kim BY, Kang SM, Kang JH, Kang SY, Kim KK, Kim KB, et al. 2020 Korean Society for the Study of Obesity Guidelines for the Management of Obesity in Korea. Journal of obesity & metabolic syndrome. 2021;30(2):81-92.

24. Rhee EJ, Kim HC, Kim JH, Lee EY, Kim BJ, Kim EM, et al. 2018 Guidelines for the Management of Dyslipidemia in Korea. Journal of lipid and atherosclerosis. 2019;8(2):78-131.

25. Arnett DK, Khera A, Blumenthal RS. 2019 ACC/AHA Guideline on the Primary Prevention of Cardiovascular Disease: Part 1, Lifestyle and Behavioral Factors. JAMA Cardiol. 2019;4(10):1043-4.

26. DeLong ER, DeLong DM, Clarke-Pearson DL. Comparing the areas under two or more correlated receiver operating characteristic curves: a nonparametric approach. Biometrics. 1988;44(3):837-45.

27. Jung KJ, Jang Y, Oh DJ, Oh BH, Lee SH, Park SW, et al. The ACC/AHA 2013 pooled cohort equations compared to a Korean Risk Prediction Model for atherosclerotic cardiovascular disease. Atherosclerosis. 2015;242(1):367-75.

28. Yang X, Li J, Hu D, Chen J, Li Y, Huang J, et al. Predicting the 10-Year Risks of Atherosclerotic Cardiovascular Disease in Chinese Population: The China-PAR Project (Prediction for ASCVD Risk in China). Circulation. 2016;134(19):1430-40.

29. Cavalcante JL, Lima JA, Redheuil A, Al-Mallah MH. Aortic stiffness: current understanding and future directions. Journal of the American College of Cardiology. 2011;57(14):1511-22.

30. Palombo C, Kozakova M. Arterial stiffness, atherosclerosis and cardiovascular risk: Pathophysiologic mechanisms and emerging clinical indications. Vascular pharmacology.

2016;77:1-7.

31. Vlachopoulos C, Aznaouridis K, Stefanadis C. Prediction of cardiovascular events and all-cause mortality with arterial stiffness: a systematic review and meta-analysis. Journal of the American College of Cardiology. 2010;55(13):1318-27.

32. Laurent S, Cockcroft J, Van Bortel L, Boutouyrie P, Giannattasio C, Hayoz D, et al. Expert consensus document on arterial stiffness: methodological issues and clinical applications. European heart journal. 2006;27(21):2588-605.

33. Sang T, Lv N, Dang A, Cheng N, Zhang W. Brachial-ankle pulse wave velocity and prognosis in patients with atherosclerotic cardiovascular disease: a systematic review and meta-analysis. Hypertension research : official journal of the Japanese Society of Hypertension. 2021;44(9):1175-85. Epub 2021/06/16.

34. Ohkuma T, Ninomiya T, Tomiyama H, Kario K, Hoshide S, Kita Y, et al. Brachial-Ankle Pulse Wave Velocity and the Risk Prediction of Cardiovascular Disease: An Individual Participant Data Meta-Analysis. Hypertension (Dallas, Tex : 1979). 2017;69(6):1045-52.

35. Umemura S, Arima H, Arima S, Asayama K, Dohi Y, Hirooka Y, et al. The Japanese Society of Hypertension Guidelines for the Management of Hypertension (JSH 2019). Hypertension research : official journal of the Japanese Society of Hypertension. 2019;42(9):1235-481.

36. Kaolawanich Y, Boonyasirinant T. Incremental prognostic value of aortic stiffness in addition to myocardial ischemia by cardiac magnetic resonance imaging. BMC Cardiovasc Disord. 2020;20(1):287.

37. Kim HL, Lim WH, Seo JB, Kim SH, Zo JH, Kim MA. Improved Prognostic Value in Predicting Long-Term Cardiovascular Events by a Combination of High-Sensitivity C-Reactive Protein and Brachial-Ankle Pulse Wave Velocity. Journal of clinical medicine.

2021;10(15).

Additional Information

Author contribution

Conceptualization: HM Kim, HL Kim

Data curation: HM Kim, TM Rhee, HL Kim

Formal analysis: HM Kim, TM Rhee, HL Kim

Investigation: HM Kim, TM Rhee, HL Kim

Methodology: HM Kim, HL Kim

Project administration: HL Kim

Resources: HL Kim

Software: HM Kim, HL Kim

Supervision: HL Kim

Validation: HM Kim, TM Rhee, HL Kim

Visualization: HM Kim, TM Rhee, HL Kim

Writing – original draft: HM Kim

Writing – review & editing: HM Kim, TM Rhee, HL Kim

Availability of data and materials

The datasets used and analyzed during the current study are available from the corresponding author on reasonable request.

Conflict of interest and sources of funding

None declared.

Ethics approval and consent to participate

The study design was approved by the local ethics committee and was conducted according to the Declaration of Helsinki.

Click here to access/download;Figure;Figure_1_1110.tif

Figure 1

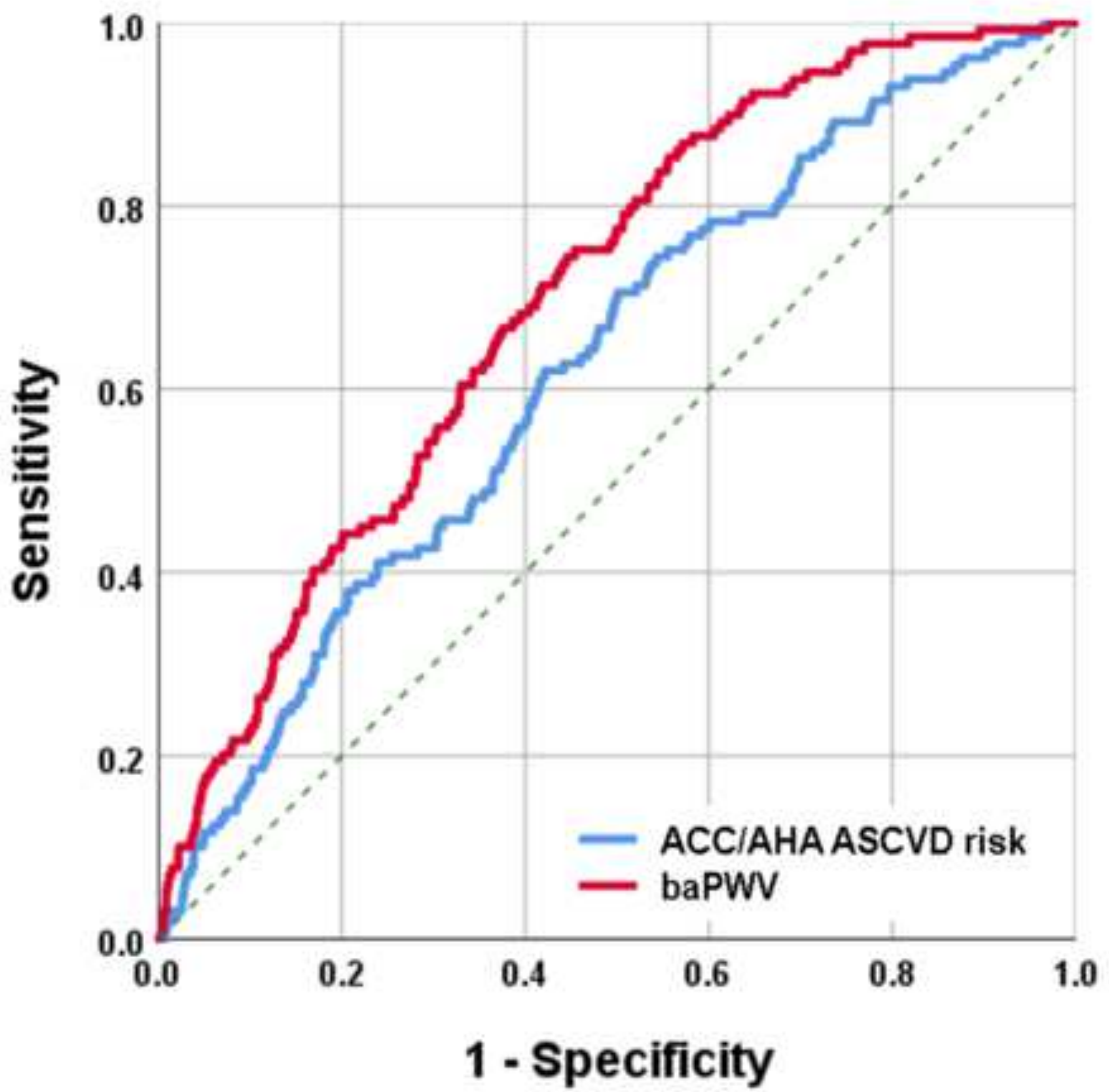

Click here to access/download;Figure;Figure_2_1110.tif

Figure 2

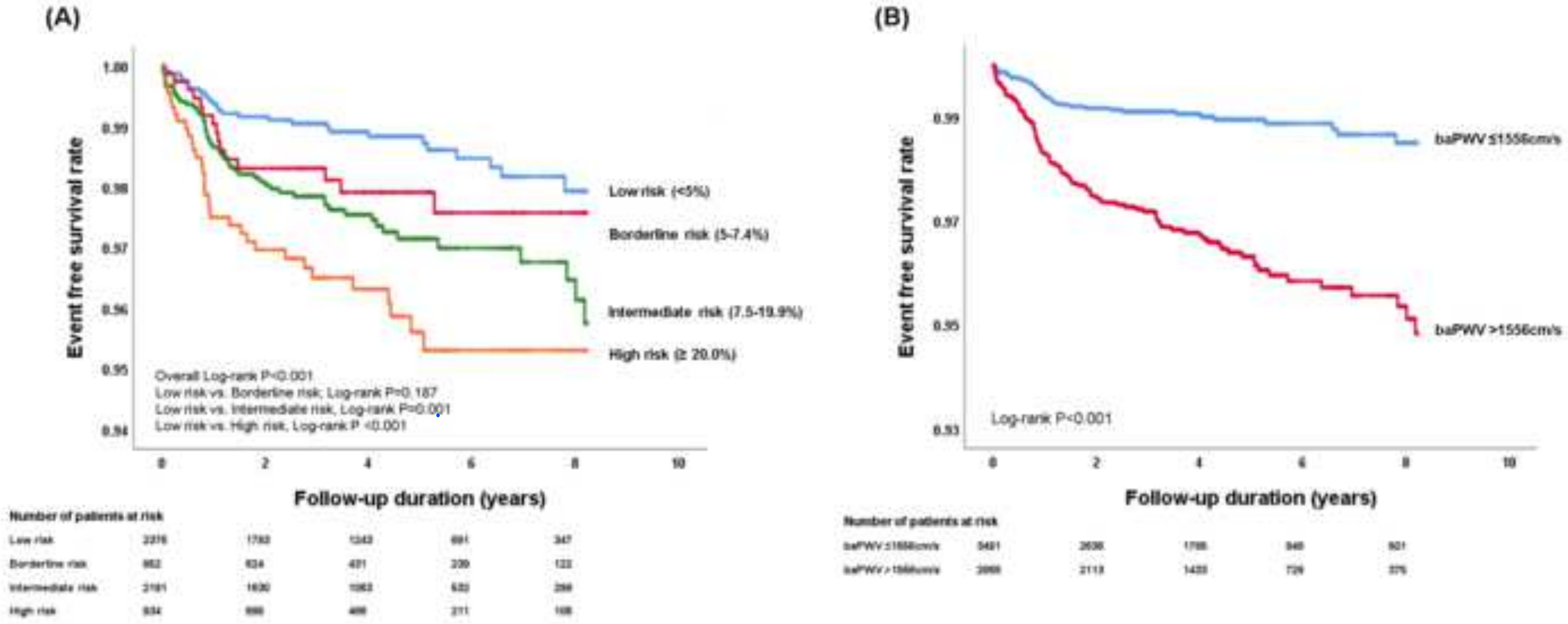

Click here to access/download;Figure;Figure_3_1110.tif

Figure 3

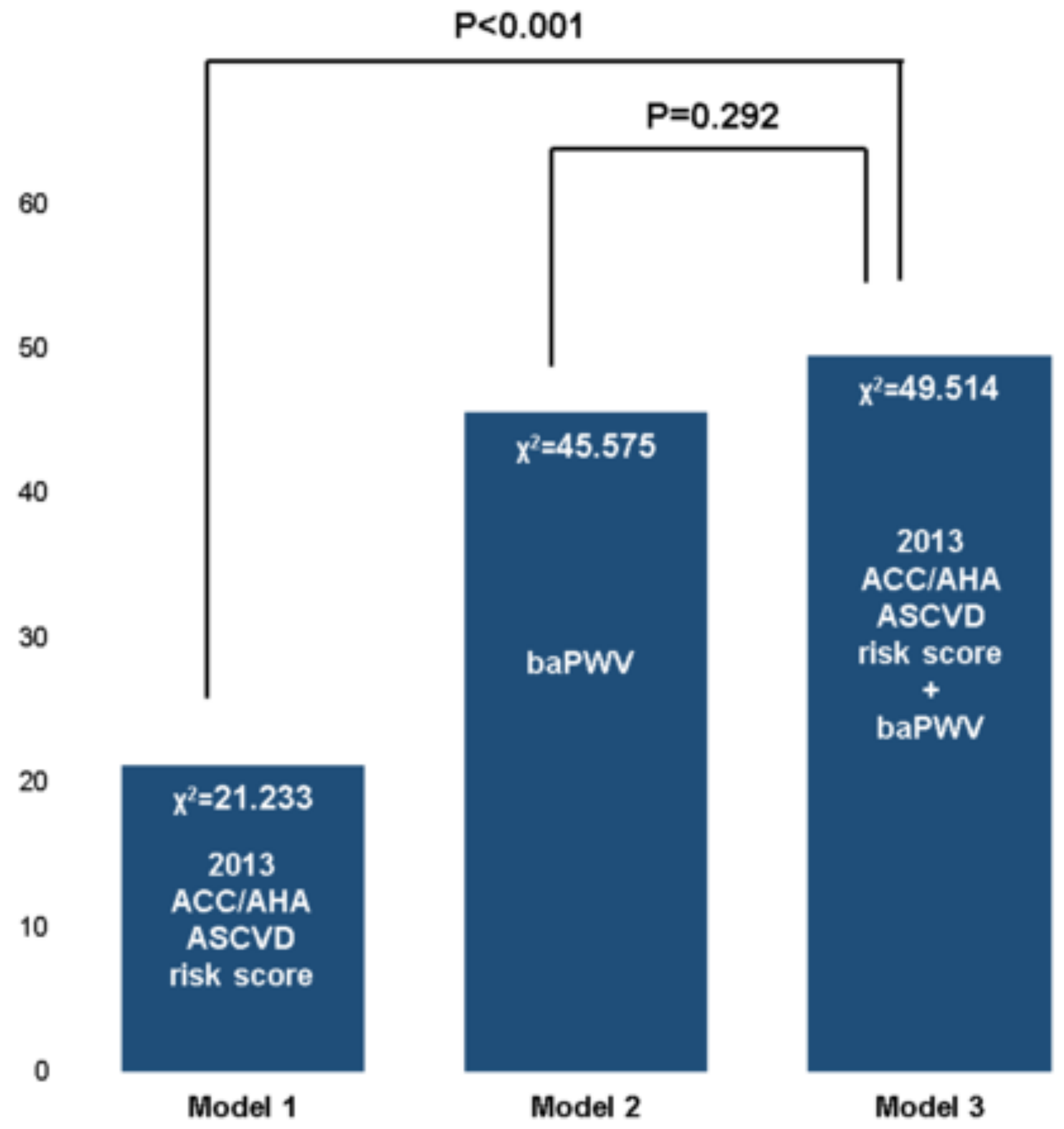

Supplement: Attachment — Submitted filename: PONE-D-22-03869_reviewer Golla.pdf [file pone.0267614.s001.pdf]
