# Supplementary material for: Integrated approach of brachial-ankle pulse wave velocity and cardiovascular risk scores for predicting the risk of cardiovascular events
Source: PLoS One. 2022 Apr 27;17(4):e0267614. doi: 10.1371/journal.pone.0267614 (PMC9045657; doi:10.1371/journal.pone.0267614)
Supplement: Attachment — Submitted filename: Response to reviewers.docx [file pone.0267614.s002.docx]

***Point-by-point Response***

***Responses to Editor Comments to the Author:***

**General comment**

Kim HM et al evaluated the Integrated Approach of Brachial-Ankle Pulse Wave Velocity and Cardiovascular Risk Scores for Predicting the Risk of Cardiovascular Events. It has shown that baPWV has further predictive value compared to the ACC/AHA risk score and have superior predictive ability when used in combination. Sample size is adequate.

I have following specific comments:

We sincerely thank Editor for his/her comments on our manuscript.

**Comment #1**

Measuring baPWV would be challenging and there is a higher chance of measurement error, need a specific vendor, well trained technician/sonographer. It is relatively costly and time consuming and may not be readily available in all centers. These limitations should be included in details in the limitation section.

**Response to comment #1**

We thank for this valuable comment. We agree with your comments that there are several limitations of PWV measurements such as requirement of specialized instrument and well-trained operators. We have added these limitations in the discussion section.

**Changes for comment #1**

We have revised the manuscript as below in Discussion section.

Discussion section: Fourthly, baPWV measurement may not be available in all centers as a part of routine clinical practice because it requires a specific vender and well-trained operators.

**Comment #2**

Need to provide more references on these bapvw, validity of aortic pwv, carotid femoral pwv and their limitations. Discussing why those are not favorite options what advantages and disadvantages.

**Response to comment #2**

We thank for this important comment. Carotid femoral PWV (cfPWV) and baPWV are the most widely used types of PWV measurement.^1^ cfPWV measurement were initiated earlier, thus it has a large amount of clinical data and is better validated. Additionally, cfPWV has been considered the gold standard measurement for large artery stiffness, because the aorta and its first branches are the most pathophysiologically important areas while considering arterial stiffness.^2^ However, measurement of cfPWV requires much skill and time for the placement of pressure-sensitive transducers on the carotid and femoral arteries, especially in obese subjects. Additionally, palpation of the femoral artery can be psychologically invasive to some subjects.^1^ To overcome these limitations, baPWV has been introduced. It has a procedural advantage of being simple to use, requiring only the wrapping of pressure cuffs. There are many clinical data showing baPWV can predictcardiovascular outcomes^3-6^. However, baPWV has been criticized for the following: including large peripheral muscular arteries, non validation of distance predicted by height, and underestimation in patients with peripheral artery stenosis, aortic aneurysm, and aortic stenosis.^7^ Therefore, specific exclusion criteria are needed in clinical research, hence we excluded those with ankle-brachial index <0.9, as generally suggested.^8^

**Changes for comment #2**

We revised manuscript as below in Discussion section.

Discussion section: Although carotid-femoral pulse(cfPWV) wave velocity is considered a gold standard non-invasive measure of arteria stiffness and has abundant clinical data.[16, 32] it has several shortcomings such as technical difficulty and inconvenience to the patients during the exam.[16] Palpation of the carotid and femoral pulses in obese patients is difficult and makes patients uncomfortable. To overcome these limitations, baPWV has been introduced. baPWV is easier and simpler to measure than cfPWV, and is widely used in Asians.[16] Although there are several criticisms of baPWV, including its inclusion of large peripheral muscular arteries, the limitation on the distance predicted by height, and underestimation in patients with peripheral artery stenosis, aortic stenosis, or aneurysm[33, 34], the usefulness of baPWV has also been validated by many clinical studies[17-20] and meta-analyses.[35, 36]

**Comment #3&#4**

I would mention the current ESC and ACC/AHA risk prediction guideline and their recommendations on the measurement of the arterial stiffness.

The ACC/AHA 2010 guidelines give CLASS III: NO BENEFIT recommendation for Specific Measures of Arterial Stiffness Measures of arterial stiffness outside of research settings are not recommended for cardiovascular risk assessment in asymptomatic adults. (Level of Evidence: C) However Measurement of Ankle-Brachial Index is CLASS IIa. Measurement of ankle-brachial index is reasonable for cardiovascular risk assessment in asymptomatic adults at intermediate risk (Level of Evidence: B). Certainly the ABI is the easiest to calculate, need little time and without cost as just need to take ankle and brachial BP.

**Response to comment #3 &#4**

Thank you for your kind comments. Both the Ankle-brachial index (ABI) and the PWV are known to be associated with prevalent cardiovascular diseases, but they are different types of risk assessment tools. The ABI is an indicator of atherosclerosis in the legs.^9^ Atherosclerosis is the narrowing of the artery by the deposition of atheromatous plaque, typically occurring in the intima layer. The PWV is an indicator of arteriosclerosis and arterial stiffness, being one of the earliest changes in vascular structure. It is a result of degenerative processes in the vessel wall, mainly in the medial layer of large elastic arteries, which is distinct from atherosclerosis.^10^

Generally, PWV measurement becomes unreliable if the circulation is disrupted on the way of pulse propagation. In this regard, bilateral measurements of the ABI are mandatory before measurement of baPWV to confirm the normal circulation of the lower limb arteries. When the ABI is lower than 0.9, peripheral artery disease (PAD) is suspected. These patients are considered to have high risk of cardiovascular disease and require closer investigation; but the value of baPWV is difficult to interpret in such situation. However, if the ABI is 0.9 or higher, PAD may be excluded.^7^ These patients have a higher risk of cardiovascular disease when the baPWV is elevated. In our study, subjects with ABI <0.9 were excluded, and baPWV ≥1,556 cm/s was an indicator of increased cardiovascular risk. In summary, PWV and ABI can be used complementarily, but it is difficult to compare the relative superiority of the two indicators.

Additionally, in the 2021 ESC guideline on cardiovascular prevention in clinical practice, which is the most recent guideline, both ABI and PWV were introduced as potential risk modifiers.^11^

**Comment #5**

I would recommend to compare the ROC between ABI and baPwv and add separate analysis with ROC using ABI and PECs in the same population. That would be more valuable if possible. You have excluded ABI < 0.9. what about to include those and compare as suggested.

**Response to comment #5**

Thank you for your valuable comment. As you recommended, we analyzed with ROC using ABI in the same population. However, as I mentioned in the previous comment, patients with ABI <0.9 should be excluded in further baPWV study due to low reliability. Therefore, all the patients in our registry had ABI ≥0.9.

The AUC of ABI (lower value between left and right) in our study was 0.55 (95% CI: 0.49-0.60, P=0.078). Pairwise comparisons of the ROC curve showed that baPWV had a more powerful predictive value in detecting ASCVD than the ABI (P <0.001 for comparison).

As we mentioned in comment 4, ABI is one of the good potential risk modifiers, but it is useful especially for patients with an abnormal ABI<0.9.^12^ Our study aimed to evaluate the prognostic value of PWV, thus new population are needed to evaluate the prognostic value of ABI.

ABI

baPWV

**General comment #1**

Abstract Methods: “A total of 6,359 patients (3,534 men and 2,825 women) aged 40-9 years without..” , it would be 79 instead of 9

**Response to general comment #1**

Thank you. We have amended the text from ‘9’ to ‘79’.

**General comment #2**

Definition of hypertension used is old “Hypertension was defined based on previous diagnosis, current use of anti-hypertensive medications or SBP/DBP ≥ 140/90 mmHg” It has changed many years back. The definition would be <130/80

**Response to general comment #2**

Thank you for your comments. As you mentioned, although the definition of hypertension in the ACC/AHA guidelines have been changed^13^, other guidelines, such as ESC, ISH, and Korean guidelines maintain the definition of hypertension as SBP/DBP≥140/90 mmHg^14-16^. Since most of the subjects in this study were Koreans, we think it is more appropriate to define hypertension as SBP/DBP ≥140/90 mmHg in this study.

**General comment #3**

Obesity defined as (body mass index ≥25kg/m2), it would be overweight and obesity defined as BMI > 30

**Response to general comment #3**

Thank you for your comment. The World Health Organization (WHO) defines obesity as BMI ≥30 kg/m^2^, but East Asians, including Koreans, have increased risk of cardiovascular disease even below a BMI of 25 kg/m^2^ and have higher body fat than Westerners at the same BMI.^17^ Therefore, in Asia pacific region, BMI of higher than 25 kg/m^2^ is considered as obesity.^14^

**General comment #4**

“baPWV could also be used as an addictive tool to more meticulously stratify cardiovascular risk in primary prevention” Not addictive tool it would be additive tool.

**Response to general comment #4**

Thank you for your comments. We changed from addictive to additive.

**General comment #5**

“The following clinical covariates were considered 137 potential confounders and included as confounding variables in the multivariable analysis”. But there are no variables mentioned

**Response to general comment #5**

Previously, we mentioned confounding variables in Table 3. We have added the co variables in the Methods section.

Methods: The following clinical covariates, including age, sex, BMI, heart rate, diabetes, hypertension, dyslipidemia, smoking, beta blockers, RAS blockers, and statin were considered potential confounders and included as confounding variables in the multivariable analysis.

***Point-by-point Response***

***Responses Reviewer#1 Comments to the Author:***

**General comment**

Brachial-Ankle Pulse Wave Velocity:" Is it a risk factor or modifier?"

It's an interesting thought provoking retrospective study. Dr.Kim's work laid a stepping stone to fill in the gap to estimate ASCVD risk in non-Caucasian population. We need more prospective studies like these to consider adding ba-PWV in ASCVD risk assessment tool.

**Response to comment**

Thank you for your kind comment.

**References**

1.	Kim HL and Kim SH. Pulse Wave Velocity in Atherosclerosis. *Front Cardiovasc Med*. 2019;6:41.

2.	Laurent S, Cockcroft J, Van Bortel L, Boutouyrie P, Giannattasio C, Hayoz D, Pannier B, Vlachopoulos C, Wilkinson I, Struijker-Boudier H and European Network for Non-invasive Investigation of Large A. Expert consensus document on arterial stiffness: methodological issues and clinical applications. *Eur Heart J*. 2006;27:2588-605.

3.	Kim HL, Jin KN, Seo JB, Choi YH, Chung WY, Kim SH, Kim MA and Zo JH. The association of brachial-ankle pulse wave velocity with coronary artery disease evaluated by coronary computed tomography angiography. *PloS one*. 2015;10:e0123164.

4.	Kim YB, Park KY, Chung PW, Kim JM, Moon HS and Youn YC. Brachial-ankle pulse wave velocity is associated with both acute and chronic cerebral small vessel disease. *Atherosclerosis*. 2016;245:54-9.

5.	Kubozono T, Miyata M, Kawasoe S, Ojima S, Yoshifuku S, Miyahara H, Maenohara S and Ohishi M. High Pulse Wave Velocity Has a Strong Impact on Early Carotid Atherosclerosis in a Japanese General Male Population. *Circulation journal : official journal of the Japanese Circulation Society*. 2017;81:310-315.

6.	Yang Y, Fan F, Kou M, Yang Y, Cheng G, Jia J, Gao L, Zhou Z, Chen D, Zhang Y and Huo Y. Brachial-Ankle Pulse Wave Velocity is Associated with the Risk of New Carotid Plaque Formation: Data from a Chinese Community-based Cohort. *Scientific reports*. 2018;8:7037.

7.	Sugawara J and Tanaka H. Brachial-Ankle Pulse Wave Velocity: Myths, Misconceptions, and Realities. *Pulse (Basel)*. 2015;3:106-13.

8.	Ato D. Pitfalls in the ankle-brachial index and brachial-ankle pulse wave velocity. *Vasc Health Risk Manag*. 2018;14:41-62.

9.	Aboyans V, Criqui MH, Abraham P, Allison MA, Creager MA, Diehm C, Fowkes FG, Hiatt WR, Jonsson B, Lacroix P, Marin B, McDermott MM, Norgren L, Pande RL, Preux PM, Stoffers HE, Treat-Jacobson D, American Heart Association Council on Peripheral Vascular D, Council on E, Prevention, Council on Clinical C, Council on Cardiovascular N, Council on Cardiovascular R, Intervention, Council on Cardiovascular S and Anesthesia. Measurement and interpretation of the ankle-brachial index: a scientific statement from the American Heart Association. *Circulation*. 2012;126:2890-909.

10.	Stone K, Fryer S, Faulkner J, Meyer ML, Heffernan K, Kucharska-Newton A, Zieff G, Paterson C, Matsushita K, Hughes TM, Tanaka H and Stoner L. Associations of lower-limb atherosclerosis and arteriosclerosis with cardiovascular risk factors and disease in older adults: The Atherosclerosis Risk in Communities (ARIC) study. *Atherosclerosis*. 2022;340:53-60.

11.	Visseren FLJ, Mach F, Smulders YM, Carballo D, Koskinas KC, Back M, Benetos A, Biffi A, Boavida JM, Capodanno D, Cosyns B, Crawford C, Davos CH, Desormais I, Di Angelantonio E, Franco OH, Halvorsen S, Hobbs FDR, Hollander M, Jankowska EA, Michal M, Sacco S, Sattar N, Tokgozoglu L, Tonstad S, Tsioufis KP, van Dis I, van Gelder IC, Wanner C, Williams B, Societies ESCNC and Group ESCSD. 2021 ESC Guidelines on cardiovascular disease prevention in clinical practice. *Eur Heart J*. 2021;42:3227-3337.

12.	Kojima I, Ninomiya T, Hata J, Fukuhara M, Hirakawa Y, Mukai N, Yoshida D, Kitazono T and Kiyohara Y. A low ankle brachial index is associated with an increased risk of cardiovascular disease: the Hisayama study. *J Atheroscler Thromb*. 2014;21:966-73.

13.	Whelton PK, Carey RM, Aronow WS, Casey DE, Jr., Collins KJ, Dennison Himmelfarb C, DePalma SM, Gidding S, Jamerson KA, Jones DW, MacLaughlin EJ, Muntner P, Ovbiagele B, Smith SC, Jr., Spencer CC, Stafford RS, Taler SJ, Thomas RJ, Williams KA, Sr., Williamson JD and Wright JT, Jr. 2017 ACC/AHA/AAPA/ABC/ACPM/AGS/APhA/ASH/ASPC/NMA/PCNA Guideline for the Prevention, Detection, Evaluation, and Management of High Blood Pressure in Adults: A Report of the American College of Cardiology/American Heart Association Task Force on Clinical Practice Guidelines. *Journal of the American College of Cardiology*. 2018;71:e127-e248.

14.	Kim BY, Kang SM, Kang JH, Kang SY, Kim KK, Kim KB, Kim B, Kim SJ, Kim YH, Kim JH, Kim JH, Kim EM, Nam GE, Park JY, Son JW, Shin YA, Shin HJ, Oh TJ, Lee H, Jeon EJ, Chung S, Hong YH and Kim CH. 2020 Korean Society for the Study of Obesity Guidelines for the Management of Obesity in Korea. *Journal of obesity & metabolic syndrome*. 2021;30:81-92.

15.	Unger T, Borghi C, Charchar F, Khan NA, Poulter NR, Prabhakaran D, Ramirez A, Schlaich M, Stergiou GS, Tomaszewski M, Wainford RD, Williams B and Schutte AE. 2020 International Society of Hypertension Global Hypertension Practice Guidelines. *Hypertension (Dallas, Tex : 1979)*. 2020;75:1334-1357.

16.	Williams B, Mancia G, Spiering W, Agabiti Rosei E, Azizi M, Burnier M, Clement DL, Coca A, de Simone G, Dominiczak A, Kahan T, Mahfoud F, Redon J, Ruilope L, Zanchetti A, Kerins M, Kjeldsen SE, Kreutz R, Laurent S, Lip GYH, McManus R, Narkiewicz K, Ruschitzka F, Schmieder RE, Shlyakhto E, Tsioufis C, Aboyans V and Desormais I. 2018 ESC/ESH Guidelines for the management of arterial hypertension: The Task Force for the management of arterial hypertension of the European Society of Cardiology and the European Society of Hypertension: The Task Force for the management of arterial hypertension of the European Society of Cardiology and the European Society of Hypertension. *Journal of hypertension*. 2018;36:1953-2041.

17.	Seo MH, Kim YH, Han K, Jung JH, Park YG, Lee SS, Kwon HS, Lee WY and Yoo SJ. Prevalence of Obesity and Incidence of Obesity-Related Comorbidities in Koreans Based on National Health Insurance Service Health Checkup Data 2006-2015. *Journal of obesity & metabolic syndrome*. 2018;27:46-52.
